# Association Between Antidiabetic Medications and Prostate-Specific Antigen Levels and Biopsy Results

**DOI:** 10.1001/jamanetworkopen.2019.14689

**Published:** 2019-11-06

**Authors:** Kerri Beckmann, Danielle Crawley, Tobias Nordström, Markus Aly, Henrik Olsson, Anna Lantz, Noor Binti Abd Jalal, Hans Garmo, Jan Adolfsson, Martin Eklund, Mieke Van Hemelrijck

**Affiliations:** 1 Translational Oncology and Urology Research, Comprehensive Cancer Centre, King’s College London, London, United Kingdom; 2 University of South Australia Cancer Research Institute, University of South Australia, Adelaide, Australia; 3 Department of Medical Epidemiology and Biostatistics, Karolinska Institutet, Stockholm, Sweden; 4 Department of Molecular Medicine and Surgery, Karolinska Institutet, Stockholm, Sweden; 5 Regional Cancer Centre, Uppsala, Sweden; 6 Department of Clinical Science, Intervention, and Technology, Karolinska Institutet, Stockholm, Sweden; 7 Unit of Epidemiology, Institute of Environmental Medicine, Karolinska Institutet, Stockholm, Sweden

## Abstract

**Importance:**

Diabetic men appear to have a lower risk of prostate cancer. Whether antidiabetic medications are protective or potentially mask prostate cancer by lowering prostate-specific antigen (PSA) levels is unclear.

**Objective:**

To examine the associations of antidiabetic medication use with (1) PSA levels, (2) frequency of PSA testing, (3) receipt of biopsy following elevated PSA results, and (4) prostate cancer detection at biopsy.

**Design, Setting, and Participants:**

Population-based cohort study using data from the Stockholm PSA and Biopsy Register. Participants were all prostate cancer–free men aged 40 to 79 years residing in Stockholm County, Sweden, between January 1, 2006, and December 31, 2015. Data were analyzed from November 2018 to March 2019.

**Exposures:**

One or more prescription for metformin, sulfonylurea, or insulin, as recorded in Sweden’s National Prescribed Drug Register.

**Main Outcomes and Measures:**

Levels of PSA following first exposure to antidiabetic medications were assessed using multivariable linear regression. Frequency of PSA testing was assessed via multivariable Poisson regression. Biopsy following elevated PSA (≥3.0 ng/mL) and prostate cancer detection at biopsy were assessed via multivariable logistic regression.

**Results:**

The cohort of 564 666 men (median [range] age, 65 [40-79] years) consisted of 4583 men initially exposed to metformin, 1104 exposed to sulfonylurea, and 978 exposed to insulin who were age matched with unexposed men (1:5). Exposed men had lower median (interquartile range) PSA levels before starting antidiabetic medications compared with unexposed men (1.2 [0.7-2.5] vs 1.6 [0.8-3.2] ng/mL). After accounting for baseline differences, PSA levels did not vary from those of unexposed men following exposure to antidiabetic medications. Frequency of PSA testing was higher for those receiving metformin (rate ratio, 1.07; 95% CI, 1.06-1.09) and sulfonylurea (rate ratio, 1.06; 95% CI, 1.03-1.08) but was lower for those receiving insulin (rate ratio, 0.79; 95% CI, 0.77- 0.81). Likelihood of biopsy after elevated PSA was lower among men receiving metformin (odds ratio, 0.87; 95% CI, 0.80-0.96) and insulin (odds ratio, 0.83; 95% CI, 0.74-0.93). There were no differences in prostate cancer detection at biopsy, regardless of PSA levels that triggered the biopsy.

**Conclusions and Relevance:**

This study’s findings do not support the hypothesis that the inverse association between diabetes and prostate cancer is mediated through antidiabetic medications lowering PSA levels to mask prostate cancer. They do suggest potential detection bias due to fewer biopsies among men receiving antidiabetic medications, which may explain the lower prostate cancer risk in men with diabetes.

## Key Points

**Question:**

What is the association between the use of antidiabetic medications and levels of prostate-specific antigen (PSA), frequency of PSA testing and biopsy, and detection of prostate cancer among diabetic men?

**Findings:**

This cohort study found no evidence that antidiabetic medications were associated with lower PSA levels or frequency of cancer detection on biopsy. However, rates of PSA testing and prostate biopsy were lower in men receiving antidiabetic medications (even when PSA was elevated).

**Meaning:**

This study suggests that detection bias due to fewer biopsies for elevated PSA among men receiving antidiabetic medications may explain some of the lower risk of prostate cancer in men with diabetes.

## Teaser

This cohort study examines the association between antidiabetic medication and prostate-specific antigen (PSA) levels, frequency of PSA testing and biopsy, and detection of prostate cancer at biopsy among Swedish men with diabetes.

## Introduction

Diabetes and prostate cancer are 2 very common conditions affecting older men. General practitioners are often involved in the diagnosis and management of both diseases.

Numerous studies have reported lower incidence of prostate cancer among men with diabetes,^1,2,3,4^ although higher risk of aggressive prostate cancer^5,6^ and poorer prognosis^7,8^ have also been noted. Reasons for the lower risk of prostate cancer are unclear, but both physiological mechanisms and detection bias have been proposed.^9^

Some studies have proposed that the lower risk of prostate cancer may be due to use of common antidiabetic medications, acting either directly or through delayed detection.^10^ Metformin, a glucose-lowering drug with antineoplastic properties commonly used as a first-line treatment for diabetes, has been suggested to be protective in the early stages of prostate cancer tumorigenesis.^11^ It is also possible that downregulation of prostate epithelial cell growth by metformin may result in lower prostate-specific antigen (PSA) levels, which mask the presence of prostate cancer or delay its detection, leading to an apparent reduced risk. Sulfonylureas and insulin analogues, which are commonly used as second- and third-line therapies for diabetes, may lead to increased insulinlike growth factor levels and hence promote prostate cancer growth and simultaneously increase PSA levels.^12,13^

To further elucidate the potential associations of antidiabetic drugs with prostate cancer development, this study was divided into 4 substudies using the same data source. The aims were to explore the potential for detection bias by examining the association of antidiabetic medication with (1) PSA levels after initial prescription; (2) frequency of PSA testing and prostate biopsy; (3) the likelihood of having a biopsy if PSA was elevated; and (4) the likelihood of prostate cancer detection at biopsy in men exposed to antidiabetic medications compared with men not exposed.

## Methods

Data were drawn from the Stockholm PSA and Biopsy Register, a population-based register of PSA tests and prostate biopsy procedures in Stockholm County, Sweden, since 2003.^14^ Data on PSA testing and biopsy results were obtained from the 3 official laboratories within the Stockholm region (Karolinska University Laboratory, Aleris, and Unilabs). Prescription dispatch dates for specific medications were determined through linkage with the National Prescribed Drug Register using Anatomical Therapeutic Chemical codes (ie, metformin, A10BA and A10BD; sulfonylurea, A10BB; and insulin, A10A), with data available since July 1, 2005. Additional covariates included age, educational level (low: <10 years, medium: 10-12 years, and high: >12 years of formal education), civil status (married or not married), family history of prostate cancer (any prostate cancer in first-degree male relatives), and comorbidity (Charlson Comorbidity Index [CCI] derived from *International Statistical Classification of Diseases and Related Health Problems, Tenth Revision (ICD-10) *codes for hospital admissions in the previous 10 years, excluding codes for diabetes; men with no hospital admission scored 0).

Ethical approval was obtained from the Stockholm regional ethics committee. All data in this study were deidentified and informed consent was waived by the ethics committee.

**Figure 1.**
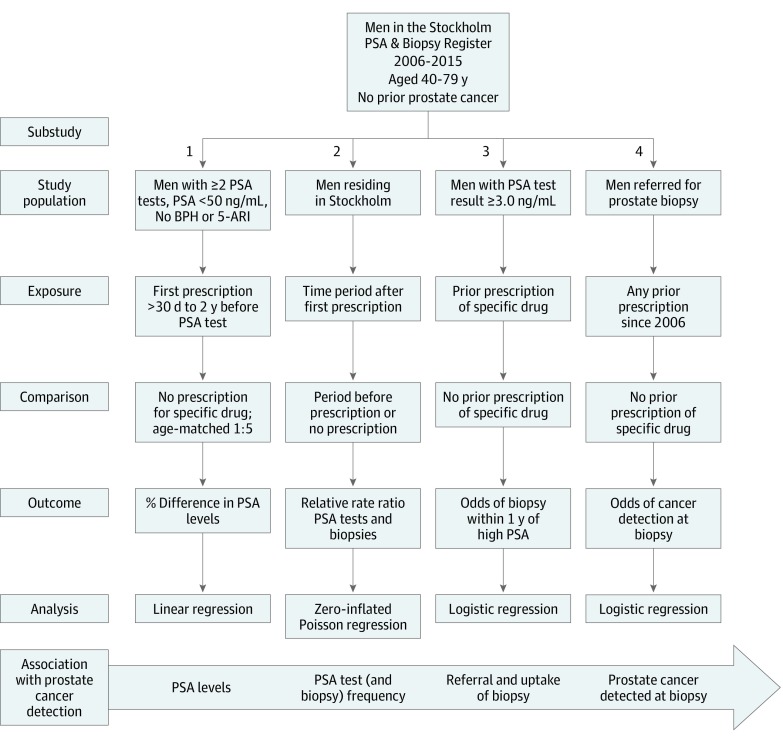
Overview of Study Designs for Each Substudy Exposures were separate classes of common antidiabetic medications (metformin, sulfonylurea, insulin) and all combined. BPH indicates benign prostatic hyperplasia; PSA, prostate-specific antigen; and 5-ARI, 5α-reductase inhibitors. To convert PSA to micrograms per liter, multiply by 1.0.

Reporting follows the Strengthening the Reporting of Observational Studies in Epidemiology (STROBE) reporting guideline for cohort studies where applicable. An overview of the 4 substudies is shown in Figure 1.

### Change in PSA Levels

Men aged 40 to 79 years between January 1, 2006, and December 31, 2015, with at least 2 PSA tests were selected, allowing for PSA levels before and after medication use to be measured. Men with a previous prostate cancer diagnosis, diagnosis of benign prostatic hyperplasia, transurethral resection of the prostate, prescription for 5α-reductase inhibitors, or serum PSA concentrations greater than 50 ng/mL (to convert to micrograms per liter, multiply by 1.0) were excluded to avoid influences of prostatic diseases or treatments.

Exposure to each class of antidiabetic medication was considered separately. Exposed men included all those who received their first prescription for that class of medication between 2 consecutive PSA test dates within 2 years before and after the date of first prescription, with 30 days or more of exposure before the second test. Five age-matched comparison men without any prior prescription for that medication who had undergone 2 PSA tests within 2 years of the respective prescription date were randomly selected via an incidence sampling approach. A 6-month run-in period from July 1 to December 31, 2005, was used to minimize misclassification of first prescription of each class of medication (ie, those with any antidiabetic medication during that period were excluded because they could not be considered newly exposed).

The association of antidiabetic medication use with PSA levels was assessed using multivariable ordinary least-squares regression, with log-transformed PSA concentration at the second test as the dependent variable, adjusted for premedication PSA (log-transformed), education, civil status, CCI, family history, test year, duration between tests, and exposure to other antidiabetic medications prior to the second PSA test. Log-level transformation of β coefficients yielded the percentage difference in PSA levels between men exposed to specific antidiabetic medications and those not exposed after accounting for differences in premedication PSA.

### Frequency of PSA Testing and Prostate Biopsies

Men living in Stockholm between 2006 and 2015 were included as a dynamic cohort. Each contributed person-time while aged 40 to 79 years, or until the date of emigration, death, or prostate cancer diagnosis. The number of PSA tests and biopsy procedures undertaken was determined for each calendar year of follow-up based on dates of tests or procedures recorded in the Stockholm PSA and Biopsy Register. For exposed men, person-time was split at the year of first antidiabetic medication prescription and coded as exposed from that year onward. Men prescribed antidiabetic medications during the run-in period (July 1, 2005, to December 31, 2005) were coded as exposed for the entire follow-up period.

Relative rate ratios comparing frequency of PSA testing and biopsy procedures in men exposed and unexposed were determined using multivariable zero-inflated Poisson regression, with adjustment for age, calendar year, education level, civil status, and family history of prostate cancer and mutual adjustment for metformin, sulfonylurea, and insulin. Additional analysis was undertaken whereby individuals prescribed antidiabetic medications at any time during follow-up were classified as exposed for the entire period, with the focus being on health behaviors in those predisposed to developing diabetes.

### Biopsy Referral and Attendance

Likelihood of a prostate biopsy following an elevated PSA was examined using multivariable logistic regression within the eligible cohort whose PSA measured 3.0 ng/mL or greater (or ≥4.0 ng/mL). If more than 1 test result was elevated, the highest PSA concentration during the study period was selected as the index PSA test. Receipt of a prostate biopsy within 12 months of that PSA test date was the outcome of interest. Previous prescriptions for metformin, sulfonylurea, or insulin were considered simultaneously as the exposures of interest. Covariates included age, year of PSA test, education, marital status, family history, and CCI.

### Prostate Cancer Detection at Biopsy

Men aged 40 to 79 years who had undergone prostate biopsies between 2006 and 2015 were selected. Confirmatory and follow-up biopsies after cancer diagnosis were excluded. The main outcome was whether prostate cancer was detected at biopsy. Men were considered exposed to antidiabetic medications if they had received at least 1 prescription 30 days or more before the date of biopsy, from July 1, 2005, onward. Associations between antidiabetic medications and positive biopsy were investigated using multivariable binary logistic regression. Models were adjusted for age, education level, civil status, CCI, family history, and PSA concentration prior to biopsy (log-transformed), and different classes of antidiabetic medication. We modeled prostate cancer detection including first biopsy only, and also all prediagnostic prostate biopsies, with adjustment for clustering due to nonindependence. Further analysis of length of time exposed to each medication was undertaken, along with subgroup analyses for specific ranges of PSA concentration triggering the biopsy.

### Statistical Analysis

Statistical methods used in this study included multivariable ordinary least-squares (linear) regression (substudy 1), Poisson regression (substudy 2), and binary logistic regression (substudies 3 and 4), with the level of statistical significance set at 2-tailed *P* < .05. All analyses were undertaken using Stata statistical software version 14 (StataCorp) from November 2018 to March 2019.

## Results

### Serum PSA Levels

**Figure 2.**
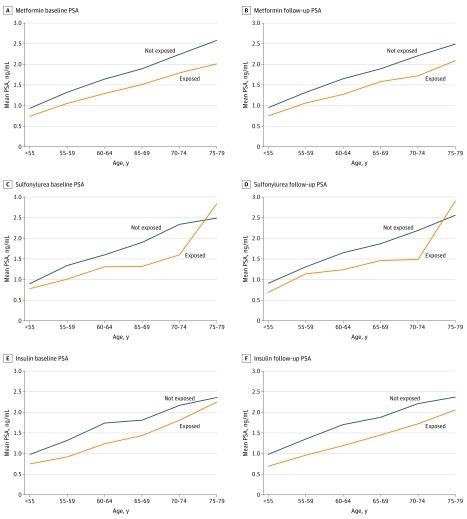
Age-Specific Prostate-Specific Antigen (PSA) Levels in Men Exposed and Not Exposed to Diabetes Medications, Before and After Initial Prescription To convert PSA to micrograms per liter, multiply by 1.0.

Four separate antidiabetic drug cohorts were identified (metformin, 4583 participants; sulfonylurea, 1104 participants; insulin, 987 participants; and first of any antidiabetic medication, 4424 participants). Characteristics of each cohort, along with their respective age-matched comparison groups, are provided in eTable 1 in the Supplement. The median (interquartile range) age at their baseline PSA test was 65.0 (59.4-70.5) years for metformin, 65.5 (59.7-71.3) years for sulfonylurea, and 66.3 (60.5-72.5) years for insulin. Median (interquartile range) PSA levels prior to first prescription were lower for exposed compared with unexposed men, for any antidiabetic use (1.2 [0.7-2.5] ng/mL vs 1.6 [0.8-3.2] ng/mL), and across all age groups for each antidiabetic medication (Figure 2).

Results of multivariable regression are shown in Table 1. No differences in postmedication PSA levels were observed for any class of medication compared with nonexposed men. Preprescription PSA level and time between PSA tests were positively associated with PSA at follow-up, while CCI of 2 or greater was associated with lower PSA levels (eTable 2 in the Supplement). No discernible patterns were evident with respect to length of exposure for any antidiabetic medications, although a significant difference was seen for 1 to 6 months of insulin use but not for longer periods.

**Table 1.** Percentage Difference in PSA Levels According to Length of Time on Antidiabetic Medications Prior to PSA Test^a^

Length of Time Using Medication Before PSA Test, mo	No. Exposed	Difference (95% CI), %	*P* Value
First antidiabetic, any			
Duration before PSA test	4424	–0.9 (–2.3 to 0.5)	.21
1-6	1741	–2.0 (–4.2 to 0.2)	.07
7-12	1433	0.2 (–1.9 to 2.4)	.86
13-18	737	–0.6 (–3.7 to 2.6)	.71
19-24	513	–0.2 (–3.8 to 3.4)	.90
Metformin			
Duration before PSA test	4583	–0.7 (–2.1 to 0.8)	.38
1-6	1799	–1.0 (–3.2 to 1.1)	.35
7-12	1491	–0.4 (–2.5 to 1.8)	.75
13-18	767	0.1 (–3.1 to 3.3)	.95
19-24	526	–1.2 (–4.9 to 2.4)	.51
Sulfonylurea			
Duration before PSA test	1104	4.1 (–0.8 to 9.1)	.10
1-6	431	5.2 (–0.7 to 11.1)	.08
7-12	366	5.5 (–0.5 to 11.5)	.07
13-18	171	0.4 (–6.1 to 6.9)	.90
19-24	136	5.0 (–3.1 to 13.2)	.22
Insulin			
Duration before PSA test	978	–6.5 (–13.8 to 0.8)	.08
1-6	404	–9.4 (–18.0 to –0.9)	.03
7-12	301	–2.0 (–10.2 to 6.3)	.64
13-18	171	–6.1 (–15.9 to 3.7)	.22
19-24	102	–3.6 (–14.7 to 7.5)	.53

Abbreviation: PSA, prostate-specific antigen.

SI conversion factor: To convert PSA to μg/L, multiply by 1.0.

^a^ Derived from multivariable linear regression for (log-transformed) PSA at follow-up, adjusted for premedication PSA (log-transformed), education, civil status, Charlson Comorbidity Index, family history of prostate cancer, test year, duration between tests and exposure to other antidiabetic medications prior to the second PSA test. Comparison groups consisted of men not previously exposed to the specific class of antidiabetic medication age matched at follow-up PSA test (5:1).

### Frequency of PSA Testing and Prostate Biopsy

The cohort description and crude rates of PSA testing and prostate biopsy are provided in eTable 3 in the Supplement. A total of 564 666 prostate cancer–free men (median [range] age, 65 [40-79] years) contributed 4 252 532 person-years of follow-up between 2006 and 2015. The mean annual rate of PSA testing was 223 per 1000, while the mean annual rate of biopsy was 6.5 per 1000.

Multivariate analyses (Table 2; eTable 4 in the Supplement) indicate increased frequency of PSA testing and biopsy procedures among men with higher education, married men, and those with a family history of prostate cancer. Rates of PSA testing were slightly higher among men prescribed metformin (relative rate ratio [RR], 1.07; 95% CI, 1.06-1.09) and sulfonylurea (RR, 1.06; 95% CI, 1.03-1.08), but lower for insulin (RR, 0.79; 95% CI, 0.77-0.81). Overall, men who received antidiabetic medications at any time during follow-up had slightly lower rates of PSA testing (RR, 0.93; 95% CI, 0.92-0.94) than those never exposed.

**Table 2.** Relative RRs for PSA Testing and Prostate Biopsy Frequency and Odds of Undergoing a Biopsy Within 12 Months of an Elevated PSA Measure According to Exposure to Antidiabetic Medications

Exposure	Frequency, RR (95% CI)^a^		Biopsy Following Elevated PSA, OR (95% CI)^b^^,^^c^	
	PSA Testing	Prostate Biopsy	≥3.0 ng/mL (n = 53 357)	≥4.0 ng/mL (n = 38 719)
Metformin	1.07 (1.06-1.09)	0.76 (0.70-0.83)	0.87 (0.80-0.96)	0.87 (0.79-0.96)
Sulfonylurea	1.06 (1.03-1.08)	0.93 (0.83-1.04)	0.88 (0.78-1.00)	0.88 (0.76-1.01)
Insulin	0.79 (0.77-0.81)	0.67 (0.60-0.75)	0.83 (0.74-0.93)	0.81 (0.71-0.92)
Any antidiabetic medication^d^	0.93 (0.92-0.94)	0.59 (0.55-0.62)	0.87 (0.80-0.96)	0.77 (0.71-0.82)

Abbreviations: OR: odds ratio; PSA, prostate-specific antigen; RR, rate ratio.

SI conversion factor: To convert PSA to μg/L, multiply by 1.0.

^a^ Rate ratios derived from zero-inflated Poisson regression models adjusted for age, calendar year, education level, marital status, family history of prostate cancer, and specific medications (simultaneously). Follow-up time for exposure was split at first prescription for the specific class of antidiabetic drug.

^b^ Odds ratios derived from binary logistic regression models for biopsy procedure within 12 months of elevated PSA level, adjusted for age group (10-year bands), year of PSA test, education, marital status, family history of prostate cancer, and Charlson Comorbidity Index score (0, 1, 2, or ≥3). Antidiabetic medications were modeled simultaneously, with a separate model for any diabetic medication.

^c^ Index PSA was the highest PSA value during follow-up if more than 1 test result was above the cutoff level.

^d^ Separate model for ever vs with never user of any diabetic medications during follow-up period.

With respect to frequency of prostate biopsy procedures, men prescribed metformin had reduced rates (RR, 0.76; 95% CI, 0.70-0.83), as did those prescribed insulin (RR, 0.67; 95% CI, 0.60-0.75). Biopsy rates did not differ for sulfonylurea. Overall, men who received any antidiabetic medication during follow-up had a significantly lower rate of prostate biopsy (RR, 0.59; 95% CI, 0.55-0.62).

### Biopsy Referral and Attendance

In all, 53 357 men had at least 1 PSA test result 3.0 ng/mL or greater, and 38 719 at least 1 test result 4.0 ng/mL or greater, of whom 36% and 43%, respectively, underwent a prostate biopsy within 12 months. Characteristics are presented in eTable 5 in the Supplement according to whether a biopsy was performed. Use of antidiabetic medications was associated with lower likelihood of biopsy following elevated PSA (Table 3; eTable 6 in the Supplement). Among men with PSA level 3.0 ng/mL or greater, adjusted odds ratios were 0.87 (95% CI, 0.80-0.96) for men who had used metformin, 0.88 (95% CI, 0.78-1.00) for men who had used sulfonylurea, and 0.83 (95% CI, 0.74-0.93) for men who had used insulin compared with no prior exposure. A similar pattern was observed among men with PSA level 4.0 ng/mL or greater.

**Table 3.** Odds Ratios for Prostate Cancer Detected at First Biopsy According to Length of Time Using Antidiabetic Medications and PSA Value Triggering Biopsy in 32 123 Participants^a^

Antidiabetic Medication	Metformin		Sulfonylurea		Insulin	
	No. Exposed	OR (95% CI)	No. Exposed	OR (95% CI)	No. Exposed	OR (95% CI)
Any previous use	1974	1.03 (0.92-1.16)	878	1.10 (0.93-1.30)	900	0.96 (0.82-1.13)
Years of previous use^b^						
<1	412	1.19 (0.96-1.47)	213	0.95 (0.71-1.27)	175	1.01 (0.73-1.41)
1 to <2	325	0.93 (0.73-1.18)	156	1.09 (0.77-1.54)	140	0.92 (0.64-1.31)
2 to <3	262	0.98 (0.75-1.27)	104	1.06 (0.72-1.56)	85	0.90 (0.57-1.41)
3 to <4	205	0.99 (0.74-1.32)	102	1.20 (0.81-1.78)	99	0.97 (0.64-1.46)
≥4	770	1.01 (0.85-1.20)	303	1.16 (0.90-1.49)	401	0.99 (0.80-1.23)
Any previous use, stratified by trigger PSA, ng/mL^c^						
<3	285	1.02 (0.72-1.44)	133	0.88 (0.53-1.46)	133	0.83 (0.51-1.36)
3 to <6	738	1.00 (0.83-1.20)	284	1.02 (0.78-1.35)	290	0.90 (0.69-1.17)
6 to <10	468	1.17 (0.94-1.45)	210	1.23 (0.91-1.66)	208	1.14 (0.85-1.53)
10 to <20	247	0.96 (0.71-1.31)	108	1.09 (0.72-1.63)	123	0.92 (0.63-1.36)
≥20	163	0.95 (0.60-1.52)	100	1.16 (0.66-2.02)	97	1.15 (0.64-2.05)

Abbreviations: OR, odds ratio; PSA, prostate specific antigen.

SI conversion factor: To convert PSA to μg/L, multiply by 1.0.

^a^ Multivariable logistic regression simultaneously adjusting for age at biopsy, log of trigger PSA, Charlson Comorbidity Index score, education level, civil status, family history of prostate cancer, and simultaneous exposure to other diabetes medications (first biopsy only).

^b^ Total years of use from first to last prescription before biopsy, assessed in a single model with participants never exposed to a specific class of antidiabetic medications as the reference.

^c^ Separate models comparing prior exposure to antidiabetic medications with no exposure, within subcategories of PSA concentration triggering referral for biopsy.

### Prostate Cancer Detection at Biopsy

Characteristics of men who underwent biopsy are shown in eTable 7 in the Supplement. A total of 32 123 men who underwent 39 160 biopsies were included. A total of 41% had prostate cancer detected at biopsy.

Results of multivariable logistic regression indicated that previous exposure to metformin, sulfonylurea, or insulin was not associated with biopsy findings (Table 3; eTable 8 in the Supplement). Results were similar when considering all biopsy events and first biopsy only. There was no indication of a dose-response association for any class of medication. Results of analyses stratified by trigger PSA concentration also showed no association at any PSA ranges.

## Discussion

This cohort study using data from a population-wide registry study found that (1) use of antidiabetic medications was not differentially associated with PSA levels, although serum PSA levels were lower than those in a comparison group of men of the same age before the initial prescription of antidiabetic medication; (2) overall frequency of PSA testing was lower among men who received any antidiabetic medication compared with those who did not, but was higher after commencing use of metformin and sulfonylurea and lower after commencing use of insulin; (3) prostate biopsy procedures were less frequent in men ever prescribed antidiabetic medications, including when PSA level was elevated; and (4) prostate cancer detection rates at biopsy did not differ among men exposed to common antidiabetic medications, regardless of PSA trigger levels.

Our findings of no difference in PSA levels after initial prescriptions of antidiabetic drugs do not support the hypothesis that the use of common antidiabetic medications leads to lower PSA levels that may mask the presence of prostate cancer in men with diabetes. While lower serum PSA levels among men with diabetes have consistently been reported,^15,16,17,18^ evidence for specific antidiabetic medications is mixed. Several reports^19,20^ indicate lower PSA levels among men taking different classes of medications, while another^21^ reported lower PSA levels only among men receiving metformin, although without a clear dose-response association. In contrast, others studies^22,23^ have reported higher PSA concentrations among men using metformin. Variation in findings may be due to differences in study design and comparison populations. For example, restricting the study population to men with diabetes^22,23^ may yield different results from population-wide studies. Except for the article by Wallner et al,^16^ none of these studies accounted for differences in PSA levels prior to medication use. Our results, which adjusted for differences in premedication PSA levels, lend support to lower PSA levels being associated with disease rather than with medication. With respect to insulin use, significant differences in postmedication PSA levels were observed for exposure within 6 months of PSA being measured. However, these findings are consistent with greater relative reductions in PSA concentration with worsening disease.^20,24^

Studies examining the frequency of PSA testing among men with diabetes are sparse, despite detection bias being a possible explanation for lower prostate cancer risk. While lower frequency of testing has been reported among obese men, who are at greater risk of developing diabetes,^25,26^ more frequent PSA testing has been reported in men with comorbid conditions, particularly cardiovascular conditions.^26,27^ For diabetes specifically, some^26,28,29^ but not all^24,30^ studies report increased frequency of PSA testing among men with diabetes. Our findings suggest that men who are likely to develop diabetes undergo PSA testing less frequently, but once prescribed metformin and sulfonylurea undergo slightly more frequent testing compared with those not receiving medications. Fowke et al^29^ have hypothesized that higher rates of PSA testing in men with specific chronic conditions may be linked to the need for routine blood testing. Our results support this theory. Reduced frequency of PSA testing after commencing use of insulin may reflect recommendations not to offer PSA testing to men with limited life expectancy due to chronic disease (eg, severe diabetes).^31^

The lower biopsy rates in men receiving antidiabetic medications may partly be explained by the lower PSA levels in these men. However, our findings also indicate that comparatively fewer men receiving antidiabetic medications undergo biopsy when PSA is elevated. We cannot determine whether this association was due to decreased referral rates or lower compliance with biopsy recommendations. There have been reports of lower compliance with biopsy recommendations among diabetic men within trial settings (eg, A Clinical Research Study to Reduce the Incidence of Prostate Cancer in Men Who Are at Increased Risk [REDUCE]^32^ and Selenium and Vitamin E Cancer Prevention Trial [SELECT],^33^ although no association was observed in the Prostate Cancer Prevention Trial [PCPT] trial^33^). However, clinicians may also be less likely to recommend biopsy owing to the increased comorbidity associated with diabetes, which may limit mens’ suitability for radical treatment.

We found no difference in prostate cancer detection rates at biopsy, which applied across different PSA trigger ranges, suggesting that use of diabetes medications is not associated with prostate cancer detection at biopsy. If lower PSA levels were masking the presence of prostate cancer, an increase in prostate cancer detection might be expected. Our results are inconsistent with reports of increased prostate cancer detection among men with diabetes or poor glycemic control.^34,35,36^ Two of the aforementioned studies, however, were undertaken in Asian populations where a positive rather than negative association between diabetes and prostate cancer risk is more prominent.^37^ Furthermore, previous studies have not specifically examined biopsy findings in relation to specific antidiabetic medications, which may ameliorate the effects of diabetes itself.

### Strengths and Limitations

Strengths of this study include its large size and population-wide coverage, with inclusion of all prescribed antidiabetic drugs, PSA testing, and biopsy procedures for men in Stockholm via linkages to high-quality registers. In contrast to previous studies, our study specifically measured differences in PSA levels with adjustment for PSA before the first prescription. This is advantageous, as associations between the medications and outcome can be more easily distinguished from associations between disease and outcome. In addition, we explored several potential mechanisms through which antidiabetic medications may be associated with detection of prostate cancer within the same cohort and time period to provide a more comprehensive assessment of the potential for detection bias.

This study also has limitations, including having access to prescription data only from July 2005 onward. We did, however, included a 6-month run-in period. Second, we did not consider all possible diabetes medications in our analyses, although metformin, sulfonylurea, and insulin are the most common medications used in Sweden for diabetes.^38^ In addition, men whose diabetes is managed through diet and lifestyle interventions alone may have been included in the unexposed group. Lack of primary care data on specific comorbidities and lifestyle factors such as BMI and smoking has limited our ability to control for confounders. Another limitation was lack of adjustment for other common medications, such as antihypertensives or lipid-lowering medications, which may potentially affect PSA levels. However, men receiving 5α-reductase inhibitors or who had undergone transurethral resection of the prostate were excluded. In addition, data on biopsy technique, number of cores, and prostate volume were not available for all men.

## Conclusions

Our findings do not support the hypothesis that the inverse association between diabetes and prostate cancer risk is mediated through use of antidiabetic medications that lower PSA and mask the presence of prostate cancer. Our findings do indicate the potential for detection bias (eg, fewer biopsies undertaken when PSA is elevated), which may explain some of the lower risk in men with diabetes.

## Notes

Supplement. **eTable 1. **Cohort Characteristics—Studying the Effects of Antidiabetic Medication on PSA Levels **eTable 2.** Full Model Results: Percent Difference in Follow-up PSA Levels Following First Exposure to Anti-diabetes Medications **eTable 3.** PSA Testing and Prostate Biopsy Rates Among Men Aged 40-79 Years Living in Stockholm County 2006-2015 **eTable 4.** Full Model Results: Zero-Inflated Poisson Regression Assessing Rates of PSA Testing and Prostate Biopsy, Among Men Aged 40-79 Years Living in Stockholm County (2006-2015) **eTable 5.** Characteristics of Men Within an Elevated PSA Test Result According to Whether They Underwent a Prostate Biopsy Within 12 Months of the Relevant PSA Test **eTable 6.** Full Model Results: Likelihood of Prostate Biopsy Within 12 Months of PSA Test Result of ≥3.0 and ≥4.0 ng/mL, Among Men Aged 40-79 Years in Stockholm County, Tested 2006-2014 **eTable 7.** Characteristics of Study Population Examining the Effects of Antidiabetic Medications on Prostate Cancer Detection at Biopsy **eTable 8.** Full Models Results: Odds Ratios for PCa Detected at Biopsy
