# Supplementary material for: Association Between Antidiabetic Medications and Prostate-Specific Antigen Levels and Biopsy Results
Source: JAMA Netw Open. 2019 Nov 6;2(11):e1914689. doi: 10.1001/jamanetworkopen.2019.14689 (PMC6865613; doi:10.1001/jamanetworkopen.2019.14689)
Supplement: Supplement. — eTable 1. Cohort Characteristics—Studying the Effects of Antidiabetic Medication on PSA Levels eTable 2. Full Model Results: Percent Difference in Follow-up PSA Levels Following First Exposure to Anti-diabetes Medications eTable 3. PSA Testing and Prostate Biopsy Rates Among Men Aged 40-79 Years Living in Stockholm County 2006-2015 eTable 4. Full Model Results: Zero-Inflated Poisson Regression Assessing Rates of PSA Testing and Prostate Biopsy, Among Men Aged 40-79 Years Living in Stockholm County (2006-2015) eTable 5. Characteristics of Men Within an Elevated PSA Test Result According to Whether They Underwent a Prostate Biopsy Within 12 Months of the Relevant PSA Test eTable 6. Full Model Results: Likelihood of Prostate Biopsy Within 12 Months of PSA Test Result of ≥3.0 and ≥4.0 ng/mL, Among Men Aged 40-79 Years in Stockholm County, Tested 2006-2014 eTable 7. Characteristics of Study Population Examining the Effects of Antidiabetic Medications on Prostate Cancer Detection at Biopsy eTable 8. Full Models Results: Odds Ratios for PCa Detected at Biopsy [file jamanetwopen-2-e1914689-s001.pdf]

Supplementary Online Content

Beckmann K, Crawley D, Nordström T, et al. Association between antidiabetic medications and prostate-specific antigen levels and biopsy results. *JAMA Netw Open*. 2019;2(11):e1914689. doi:10.1001/jamanetworkopen.2019.14689

eTable 1. Cohort Characteristics—Studying the Effects of Antidiabetic Medication on PSA Levels

eTable 2. Full Model Results: Percent Difference in Follow-up PSA Levels Following First Exposure to Anti-diabetes Medications

eTable 3. PSA Testing and Prostate Biopsy Rates Among Men Aged 40-79 Years Living in Stockholm County 2006-2015

eTable 4. Full Model Results: Zero-Inflated Poisson Regression Assessing Rates of PSA Testing and Prostate Biopsy, Among Men Aged 40-79 Years Living in Stockholm County (2006-2015)

eTable 5. Characteristics of Men Within an Elevated PSA Test Result According to Whether They Underwent a Prostate Biopsy Within 12 Months of the Relevant PSA Test

eTable 6. Full Model Results: Likelihood of Prostate Biopsy Within 12 Months of PSA Test Result of ≥3.0 and ≥4.0 ng/mL, Among Men Aged 40-79 Years in Stockholm County, Tested 2006-2014

eTable 7. Characteristics of Study Population Examining the Effects of Antidiabetic Medications on Prostate Cancer Detection at Biopsy

eTable 8. Full Models Results: Odds Ratios for PCa Detected at Biopsy

This supplementary material has been provided by the authors to give readers additional information about their work.

© 2019 Beckmann K et al. *JAMA Network Open.*

eTable 1. Cohort characteristics - studying the effects of antidiabetic medication on PSA levels

Factors		Any medication		Metformin		Sulfonylurea		Insulin	
		Not exposed	Exposed	Not exposed	Exposed	Not exposed	Exposed	Not exposed	Exposed
Total no.		22097	4424	22899	4583	5524	1104	4872	978
		median (IQR)	median (IQR)	median (IQR)	median (IQR)	median (IQR)	median (IQR)	median (IQR)	median (IQR)
Follow-up PSA	ng/mL	1.6 (0.8-3.4)	1.3 (0.7-2.7)	1.6 (0.8-3.5)	1.3 (0.7-2.7)	1.6 (0.8-3.2)	1.2 (0.6-2.4)	1.7 (0.8-3.5)	1.2 (0.7-2.4)
Baseline PSA	ng/mL	1.6 (0.8-3.2)	1.2 (0.7-2.5)	1.6 (0.8-3.3)	1.2 (0.7-2.5)	1.6 (0.8-3.4)	1.2 (0.7-2.7)	1.6 (0.8-3.3)	1.2 (0.6-2.4)
Time Interval	months	12 (6-18)	13 (9-21)	12 (6-18)	13 (8-21)	12 (6-18)	14 (10-22)	12 (6-17)	15 (10-22)
		No. %	No. %	No. %	No. %	No. %	No. %	No. %	No. %
Age (years)	40-49	904 (4)	179 (4)	869 (4)	173 (4)	222 (4)	21 (4)	213 (4)	40 (4)
	50-59	5507 (25)	1101 (25)	5682 (25)	1129 (25)	1310 (24)	263 (24)	1084 (22)	218 (22)
	60-69	10799 (49)	2150 (49)	11086 (48)	2214 (48)	2694 (49)	538 (49)	2359 (48)	473 (48)
	70-79	4887 (22)	994 (22)	5262 (23)	1067 (23)	1293 (24)	262 (24)	1216 (25)	247 (25)
CCI	0	18559 (84)	3348 (76)	19181 (84)	3505 (76)	4613 (84)	827 (75)	4018 (82)	613 (63)
	1	1759 (8)	523 (12)	1850 (8)	544 (12)	428 (8)	136 (12)	436 (9)	142 (15)
	2+	1779 (8)	553 (13)	1868 (8)	534 (12)	483 (9)	141 (13)	418 (9)	22 (23)
Married	No	7468 (34)	1737 (39)	7719 (34)	1803 (39)	1893 (34)	448 (41)	1662 (34)	413 (42)
	Yes	14629 (66)	2687 (61)	15180 (66)	2780 (61)	3631 (66)	656 (59)	3210 (66)	565 (58)
Education	Low	8180 (37)	2082 (47)	8465 (37)	2185 (48)	2091 (38)	540 (49)	1790 (37)	481 (49)
	Medium	7562 (34)	1387 (31)	7861 (34)	1442 (32)	1895 (34)	337 (31)	1612 (33)	304 (31)
	High	6158 (28)	892 (20)	6368 (28)	889 (19)	1484 (27)	211 (19)	1417 (29)	177 (18)
	Missing	197 (1)	63 (1)	205 (1)	67 (1)	54 (1)	16 (1)	53 (1)	16 (2)
Family history Pca	No	19100 (86)	3968 (90)	19782 (86)	4106 (90)	4740 (86)	1014 (92)	4175 (86)	890 (91)
	Yes	2997 (14)	456 (10)	3117 (14)	477 (10)	784 (14)	90 (8)	697 (14)	88 (9)
Prior medications	Metformin	-	3918 (89)	-	4583 (100)	-	785 (71)	-	713 (73)
	SU	-	595 (13)	-	807 (17)	-	1104 (100)	-	388 (40)
	Insulin	-	423 (10)	-	470 (10)	-	127 (12)	-	978 (100)

CCI: Charlson Comorbidity Index; IQR: interquartile range; PSA: prostate specific antigen

© 2019 Beckmann K et al. *JAMA Network Open.*

eTable 2. Full model results: percent difference in follow-up PSA levels following first exposure to anti-diabetes medications

	**First antidiabetic drug (any)** No. exposed=4424[a]				**Metformin** No. exposed=4583[a]				**Sulfonylurea** No. exposed=1104[a]				**Insulin** No. exposed=987[a]			
**Factors:**	**% diff**	**95% CI**		**p**	**% diff**	**95% CI**		**p**	**% diff**	**95% CI**		**p**	**% diff**	**95% CI**		**p**
**Exposed to drug**	-0.9	-2.3	0.5	0.210	-0.7	-2.1	0.8	0.383	4.1	-0.8	9.1	0.101	-6.5	-13.8	0.8	0.082
% change baseline PSA	0.93	0.92	0.94	0.000	0.93	0.92	0.94	0.000	0.93	0.92	0.95	0.000	0.91	0.89	0.93	0.000
Interval (months)	4.9	4.0	5.6	0.000	5.1	4.3	5.8	0.000	4.0	2.6	5.3	0.000	4.6	3.0	6.2	0.000
CCI=0	ref	-	-	-	ref	-	-	-	ref	-	-	-	ref	-	-	-
1	0.0	-1.9	1.8	0.993	0.2	-1.6	2.0	0.862	-1.1	-4.7	2.6	0.557	1.1	-3.4	5.8	0.637
2+	-7.9	-10.6	-5.1	0.000	-9.1	-11.9	-6.1	0.000	-9.0	-14.0	-3.8	0.001	-6.8	-12.6	-0.6	0.031
Year of follow-up test	-0.3	-0.5	-0.1	0.014	-0.3	-0.5	-0.1	0.002	-0.2	-0.7	0.2	0.239	-0.1	-0.6	0.4	0.630
Married	-0.7	-1.8	0.5	0.256	-0.9	-1.9	0.3	0.130	1.1	-1.1	3.4	0.334	-0.2	-2.7	2.5	0.904
Education: Low	ref	-	-	-	ref	-	-	-	ref	-	-	-	ref	-	-	-
Medium	0.4	-0.9	1.6	0.556	0.4	-0.8	1.6	0.522	-0.1	-2.4	2.2	0.912	-1.3	-4.1	1.7	0.403
High	0.6	-0.8	1.9	0.434	0.6	-0.7	2.0	0.369	0.3	-2.5	3.2	0.813	-0.6	-3.3	2.2	0.691
Family history of PCa	-0.2	-1.9	1.5	0.918	0.2	-1.5	1.9	0.820	1.0	-1.9	4.1	0.496	1.5	-2.0	5.2	0.392
Prior exposure to:																
Metformin	-	-	-	-	-	-	-	-	-6.3	-11.6	-0.7	0.029	3.2	-4.8	11.8	0.442
Sulfonylurea	-	-	-	-	0.1	-4.0	4.3	0.975	-	-	-	-	1.6	-4.8	8.4	0.636
Insulin	-	-	-	-	-1.9	-5.2	1.6	0.283	0.5	-6.9	8.5	0.895	-	-	-	-

CI: confidence interval; PCa: Prostate cancer; PSA: prostate specific antigen; ref: reference group.
% Diff: per cent difference in PSA - Derived from multivariable linear regression for (log-transformed) PSA at follow-up
[a] Comparison groups consisted of men not previously exposed to the specific class of antidiabetic medication age matched at follow-up PSA test (5:1)

© 2019 Beckmann K et al. *JAMA Network Open.*

eTable 3. PSA testing and prostate biopsy rates among men aged 40-79 years living in Stockholm County 2006-2015

		No. (%) persons	Follow-up person years[a]	Avg. annual rate PSA test (per 1000)	Avg. annual rate biopsy (per 1000)
Total (persons)		564,666 (100)	4,252,532	223	8.7
Age (years)[b]	40-49	168,372 (38)	1,627,514	65	0.5
	50-59	119,683 (27)	1,181,781	225	7.2
	60-69	100,909 (23)	962,944	386	21.0
	70-79	48,689 (11)	472,494	443	17.0
Education level	Low	98,803 (18)	756,608	224	8.8
	Medium	228,219 (41)	1,749,589	219	8.6
	High	227,261 (41)	1,693,889	227	9.2
Married	No	245,464 (47)	1,949,796	178	6.8
	Yes	299,202 (53)	2,302,736	261	10.5
Family history PCa	No	516,732 (92)	3,865,063	215	8.2
	Yes	47,934 (8)	387,469	304	15.3
Metformin use	Yes	35, 373 (6)	147,047	285	8.7
Sulfonylurea use	Yes	18,411 (3)	68501	293	8.9
Insulin use	Yes	24,055 (4)	101,548	209	6.5
Any antidiabetic drugs[b]	Yes	45,230 (8)	320,139	212	8.4

PCa: prostate cancer; PSA: prostate specific antigen

[a] includes all follow-up time while aged 40-79yrs between year 2006 to2015

[b] age distribution in 2012

© 2019 Beckmann K et al. *JAMA Network Open.*

eTable 4. Full Model Results: Zero-inflated Poisson regression assessing rates of PSA testing and prostate biopsy, among men aged 40-79 years living in Stockholm County (2006-2015).

Factors	PSA testing		Prostate Biopsy	
	Rate ratio	99% CI	Rate ratio	99% CI
Age (years)	1.015	1.014-1.015	0.922	0.918-0.929
Calendar year	0.996	0.995- 0.997	1.004	0.995-1.013
Education level (reference: low)	1.00	-	1.00	-
Medium	1.19	1.15-1.17	1.14	1.10-1.19
High	1.33	1.31-1.34	1.23	1.16-1.30
Married (reference: Not married)	1.15	1.18-1.20	1.19	1.14-1.28
Family hist. of PCa (reference: None)	1.20	1.18-1.21	1.27	1.19-1.36
Metformin use[a]	1.07	1.06-1.09	0.76	0.70-0.83
Sulfonylurea use[a]	1.06	1.03-1.08	0.93	0.83-1.04
Insulin use[a]	0.79	0.77-0.81	0.67	0.60-0.75
Ever any antidiabetic medication[b]	0.93	0.92-0.94	0.59	0.55-0.62

CI: confidence interval; PCa: Prostate cancer; PSA: prostate specific antigen
Rate Ratios derived from Zero-inflated Poisson regression models adjusted for all covariates listed simultaneously,
[a] Time split at first prescription for specific class of antidiabetic drug, with simultaneous adjustment for prior exposure to other antidiabetic medication (separate models)
[b] 'ever prescribed antidiabetic medications' was defined as any exposure to any class of antidiabetic medication during follow-up derived. Analysed in a separate model with adjustment for the same covariates except other diabetes medications.

© 2019 Beckmann K et al. *JAMA Network Open.*

eTable 5. Characteristics of men within an elevated PSA test result according to whether they underwent a prostate biopsy within 12 months of the relevant PSA test

PSA result:	PSA≥3ng/mL		PSA≥4ng/mL	
	No biopsy <12m	Biopsy <12m	No biopsy <12m	Biopsy <12m
	No. (%)	No. (%)	No. (%)	No. (%)
Total	34232 (64)	19125 (36)	22229 (57)	16056 (43)
Age group				
40-49	1173 (3)	388 (2)	617 (3)	310 (2)
50-59	5218 (15)	4256 (22)	2897 (13)	3408 (21)
60-69	13079 (38)	10429 (54)	8127 (37)	8914 (54)
70-79yrs	14762 (43)	4052 (21)	10588 (47)	3858 (23)
Education level				
low	7795 (23)	3484 (18)	5360 (24)	3110 (19)
medium	1366 (40)	7728 (40)	8836 (40)	6682 (41)
high	12278 (39)	7793 (41)	7812 (35)	6587 (40)
missing	493 (1)	120 (1)	331 (1)	111 (1)
CCI				
0	26202 (77)	16627 (87)	16637 (75)	14245 (86)
1	3668 (11)	1397 (7)	2489 (11)	1242 (8)
2	1526 (4)	472 (2)	1081 (5)	422 (3)
3+	2836 (8)	629 (3)	2022 (9)	581 (4)
Married				
No	13585 (40)	6847 (36)	8827 (40)	5929 (36)
Yes	20647 (60)	12278 (64)	13402 (60)	10561 (64)
Family hist. PCa				
No	30660 (90)	15954 (83)	19906 (90)	13797 (84)
yes	3572 (10)	3171 (17)	2232 (10)	2693 (16)
Metformin				
No	31481 (92)	18029 (94)	20409 (92)	15543 (94)
Yes	2751 (8)	1096 (6)	1820 (8)	947 (6)
Sulfonylurea				
No	32740 (96)	18640 (97)	21216 (95)	16061 (97)
Yes	1492 (4)	485 (3)	1013 (5)	429 (3)
Insulin				
No	32725 (96)	18622 (97)	21225 (95)	16056 (97)
Yes	1507 (4)	503 (3)	1004 (5)	434 (3)

CCI: Charlson Comorbidity Index; PCa: prostate cancer; PSA: prostate specific antigen

Index PSA = highest PSA value during follow-up

© 2019 Beckmann K et al. *JAMA Network Open.*

eTable 6. Full Model results: Likelihood of prostate biopsy within 12 months of PSA test result of ≥3.0 and ≥4.0 ng/mL, among men aged 40-79 years in Stockholm County, tested 2006-2014

Factors	Total PSA ≥3.0 ng/mL n=53,357		Total PSA≥4.0 ng/mL n=38719	
	**Odds ratio**	**95% CI**	**Odds ratio**	**95% CI**
Prior exposure to:				
Metformin	0.87	0.80-0.96	0.87	0.79-0.96
Sulfonylurea	0.88	0.78-1.00	0.88	0.76-1.01
Insulin	0.83	0.74-0.93	0.81	0.71-0.92
Age 40-49	0.39	0.35-0.44	0.43	0.37-0.49
50-59	0.96	0.91-1.01	1.00	0.95-1.07
60-69 (reference)	1.00	-	-	-
70-79yrs	0.39	0.37-0.41	0.37	0.36-0.39
Education (reference: Low)	1.00	-	1.00	-
medium	1.12	1.06-1.17	1.12	1.06-1.19
high	1.14	1.08-1.20	1.13	1.07-1.20
Married (reference: not married)	1.18	1.14-1.17	1.18	1.13-1.23
Family history of PCa	1.51	1.43-1.60	1.45	1.37-1.55
CCI (reference: 0)	1.00	-	-	-
1	0.72	0.67-0.77	0.70	0.65-0.76
2	0.59	0.54-0.67	0.57	0.50-0.63
3+	0.49	0.44-0.53	0.47	0.42-0.52

Odd ratio derived from multivariable logistic regression model of biopsy procedure within 12 months of elevated PSA result, adjusted for age group(10yr bands) , year of test, education, marital status, family history of PCa, Charlson comorbidity score (0,1,2,3+).

CI: confidence interval; PCa: Prostate cancer; PSA: prostate specific antigen

Index PSA = highest PSA value during follow-up if more than 1 test result over the cut-off

© 2019 Beckmann K et al. *JAMA Network Open.*

eTable 7. Characteristics of study population examining the effects of antidiabetic medications on prostate cancer detection at biopsy

**Characteristics**		**No cancer present**	**Cancer present**
Total	No. %	19060 (59)	13063 (41)
Age at first biopsy	Mean years (SD)	64 (7)	66 (7)
Trigger PSA	Median ng/mL (IQR)	4.9 (3.4-7.3)	6.4 (4.3-11)
		No. %	No. %
Civil status	Not married	6735 (35)	4679 (36)
	Married	12325 (65)	8384 (64)
Education level	Low	3326 (17)	2527 (19)
	Medium	7503 (39)	5329 (41)
	High	8086 (42)	5083 (39)
	missing	145 (1)	124 (1)
Family history of PCa	No	16484 (86)	10496 (80)
	Yes	2576 (14)	1295 (10)
CCI score at first biopsy	0	16586 (87)	10901 (83)
	1	1346 (7)	1088 (8)
	2	526(3)	446 (3)
	3+	602 (3)	628 (5)
Prior exposure to:	Metformin	1113 (5.8)	861 (6.6)
	Sulfonylurea	471 (2.5)	407 (3.1)
	Insulin	499 (2.6)	401 (3.1)

CCI: Charlson Comorbidity Index; IQR: interquartile range; PCa: Prostate cancer; PSA: prostate specific antigen; SD standard deviation

© 2019 Beckmann K et al. *JAMA Network Open.*

eTable 8. Full models results: Odds ratios for PCa detected at biopsy

Factors	First prostate biopsy n=30,821		All biopsies pre-diagnosis[a] n=37,769	
	**OR**	**95% CI**	**OR**	**95% CI**
**Age at biopsy (years)**	1.02	1.02-1.03	1.03	1.02-1.03
**Log PSA**	1.98	1.92-2.05	1.75	1.70-1.80
**Education level**				
Low	1.00	reference	1.00	reference
Medium	1.06	0.98-1.13	1.03	0.96-1.10
High	0.98	0.91-1.05	0.96	0.90-1.02
**Married**				
No	1.00	reference	1.00	reference
Yes	1.00	0.95-1.05	0.99	0.94-1.04
**Family history of PCa**				
No	1.00	reference	1.00	reference
Yes	1.73	1.62-1.85	1.67	1.58-1.80
**Comorbidity** CCI score				
0	1.00	reference	1.00	reference
1	1.09	1.00-1.20	1.15	1.05-1.25
2	1.07	0.93-1.24	1.10	0.96-1.27
3+	1.28	1.13-1.46	1.32	1.17-1.49
**Metformin** use prior to biopsy	1.03	0.92-1.16	1.03	0.92-1.15
**Sulfonylurea** use prior to biopsy	1.10	0.93-1.30	1.14	0.97-1.34
**Insulin** use prior to biopsy	0.96	0.82-1.13	0.98	0.84-1.14

CCI: Charlson comorbidity index; CI: confidence interval; PCa: Prostate cancer; PSA: prostate specific antigen.
Multivariable logistic regression simultaneously adjusted for age at biopsy, log (trigger PSA), CCI, education level, civil status, family history of PCa, and any prior exposure to other diabetes medications.
[a]Robust estimates accounting for non-independence between men who had undergone more than 1 prostate biopsy.

© 2019 Beckmann K et al. *JAMA Network Open.*
